# Potential Role of mRNA in Estimating Postmortem Interval: A Systematic Review

**DOI:** 10.3390/ijms25158185

**Published:** 2024-07-26

**Authors:** Vincenzo Cianci, Cristina Mondello, Daniela Sapienza, Maria Cristina Guerrera, Alessio Cianci, Annalisa Cracò, Fausto Omero, Vittorio Gioffrè, Patrizia Gualniera, Alessio Asmundo, Antonino Germanà

**Affiliations:** 1 Department of Biomedical and Dental Sciences and Morphofunctional Imaging, Section of Legal Medicine, University of Messina, Via Consolare Valeria, 1, 98125 Messina, Italy; mondelloc@unime.it (C.M.); patrizia.gualniera@unime.it (P.G.); alessio.asmundo@unime.it (A.A.); 2 Zebrafish Neuromorphology Lab, Department of Veterinary Sciences, University of Messina, 98168 Messina, Italy; mariacristina.guerrera@unime.it (M.C.G.); agermana@unime.it (A.G.); 3 Department of Cardiovascular Medicine, Fondazione Policlinico Universitario A. Gemelli-IRCCS, Largo A. Gemelli 8, 00168 Rome, Italy; alessiocianci.1998@gmail.com; 4 Diagnostic and Interventional Radiology Unit, Department of Biomedical Sciences and Morphological and Functional Imaging, University Hospital Messina, 98125 Messina, Italy; annalisacraco@hotmail.it; 5 Medical Oncology Unit, Department of Human Pathology “G. Barresi”, University of Messina, 98125 Messina, Italy; faustoomero@hotmail.it; 6 Department of Otorhinolaryngology-Head and Neck Surgery, IRCCS San Raffaele, Vita-Salute San Raffaele University, Via Olgettina 60, 20132 Milan, Italy; vittoriogioffre@outlook.it

**Keywords:** PMI estimation, postmortem interval estimation, RNA degradation, mRNA degradation, forensic pathology, forensic sciences

## Abstract

Although the postmortem interval estimation still represents one of the main goals of forensic medicine, there are still several limitations that weigh on the methods most used for its determination: for this reason, even today, precisely estimating the postmortem interval remains one of the most important challenges in the forensic pathology field. To try to overcome these limitations, in recent years, numerous studies have been conducted on the potential use of the mRNA degradation time for reaching a more precise post mortem interval (PMI) estimation. An evidence-based systematic review of the literature has been conducted to evaluate the state of the art of the knowledge focusing on the potential correlation between mRNA degradation and PMI estimation. The research has been performed using the electronic databases PubMed and Scopus. The analysis conducted made it possible to confirm the potential applicability of mRNA for reaching a more precise PMI estimation. The analysis of the results highlighted the usefulness of some mRNAs, such as β-actin and glyceraldehyde-3-phosphate dehydrogenase (GAPDH) mRNA, especially in short time frames, within a few hours or days of death. The matrices on which these analyses were conducted were also analyzed, resulting in less exposure to the external environment, including the heart, brain, and dental pulp. The major limitations were also reported, including the short time intervals analyzed in most of the articles, the lack of mathematical models, and the failure to report the error rate between the mRNA degradation time and PMI. Given the still small number of published articles, the lack of globally recognized standardized methods, and the numerous techniques used to evaluate the mRNA degradation times, numerous and larger studies are still necessary to reach more solid and shared evidence.

## 1. Introduction

The estimation of the time since death is one of the main tasks of the forensic pathologist. In this context, both forensic practitioners and researchers refer to the so-called postmortem interval (PMI), defined as the time interval between an individual’s death and the discovery of their body [1]. The PMI has relevant implications that impact both the civil and criminal fields; for example, it plays a fundamental role in homicidal cases in which a wrong estimate can determine heavily negative effects on the investigation outcomes.

Nevertheless, despite the importance of a correct PMI estimation, currently, there are no methods that allow its exact determination [2].

In forensic practice, PMI analysis belongs to a specific branch called thanatology, a discipline that concerns the analysis of the macroscopic and microscopic changes of the body after death [3].

Several researchers suggested classifying the (PMI) into different stages based on time, especially referring to the early and late stages [3]. Particularly, some authors described the early postmortem (ePMI) interval as that falling within 24–36 h after death, in which there are supravital reactions characterized by metabolic processes determining tissue reactions (i.e., livor mortis, rigor mortis) [4].

The late PMI is instead referred to as the period following the first 24–36 h, in which the decomposition processes begin [4]. For this reason, the estimation of the late PMI is affected by several limitations and cannot be accurate at the present time. 

It is known that, in forensic practice, the methods mainly used to estimate the ePMI are represented by the classic triad of postmortem changes, made up of the thermometric method based on the application of the Henssge nomogram; the evaluation of rigor mortis, with reference to the maneuver of reversal of cadaveric rigidity; and the analysis of the main hypostases’ findings [5]. Nevertheless, the effectiveness of these methods is limited by both intrinsic (i.e., body mass, the body’s surface area, and the cause of death) and extrinsic (i.e., environment temperature and humidity) confounding factors, capable of influencing their reliability, especially in cases of postmortem intervals exceeding 24–48 h [6,7,8,9,10].

To overcome the difficulties in PMI estimation, in recent years, research evidence has highlighted the usefulness of the integration of forensic science with other disciplines (i.e., entomology, microbiology) [9]. Particularly, forensic entomology is a branch showing a constant increase in the number of scientific papers and can be considered an important support in PMI estimation [10]. In fact, it is routinely used in death investigations [10]. Furthermore, the analysis of the microbe’s succession in different parts/tissues of the body occurring after death, better known as thanatomicrobiome, is reported as a potentially great tool for PMI estimation [10].

Furthermore, metabolomics and the analysis of the degradation patterns of DNA, RNA, and proteins are gaining ground due to the first promising but still inaccurate results of the few articles present in the literature [11,12,13,14,15,16,17]. Among these, RNA degradation pattern analysis has received particular attention [12,14,17].

Despite this, it is known that RNAs, like other biomolecules, are influenced by various factors, which make their analysis more complex or even ineffective [12]. In fact, if temperature plays a fundamental role among the major limitations in the application of the traditional tools used for PMI estimation (i.e., Hessnge nomogram), there are many more factors that can influence the degradation patterns of these macromolecules [6,12]. Among these, the type of tissue on which a particular RNA is observed, or even the body mass index (BMI), can cause marked alterations in RNA degradation [17].

A further limitation lies in the possibility of standardizing specific RNA degradation patterns in animal models, which have always represented the first experimental models, and then transferring them to humans [12].

In the literature, different classes of RNA, each playing different roles, have been described [18]. In particular, RNAs can be distinguished into several categories: mRNA, ncRNA-containing microRNAs (miRNAs), ribosomal RNAs (rRNA), non-coding RNAs (ncRNA), PIWI-interacting RNAs (piRNAs), medium-length RNAs (snoRNAs), and longer transcripts (lcnRNAs) [18]. More simply, RNAs can be differentiated into two broad categories, which are represented by coding RNA and non-coding RNA. In any case, mRNA, microRNA, and rRNA are considered to be the most represented in eukaryotic organisms [18].

Furthermore, it is important to underline that, in the literature, the analysis of the postmortem variation in the expression of mRNA molecules has allowed us to identify both its reduction, which is due to degradation processes, and its increase; this variation is closely related to the processes in which mRNAs intervene [19]. In particular, even very substantial increases have been described in those mRNA molecules encoded for proteins involved in apoptosis and cell death processes [19].

Undoubtedly, one of the factors capable of stimulating apoptotic processes and programmed cell death (both intrinsic and extrinsic pathways) is represented by cellular anoxia, secondary to the cessation of blood flow [20]. In particular, among the numerous effectors involved in these processes, Caspase-3 (Casp3), transformation-related protein 53 (Trp53), and BCL2-associated X protein (BAX) are among the most analyzed [20]. Noshi et al. [21] conducted a study on murine liver tissue, describing their increase within the first 24 h. Subsequently, these results were confirmed in other studies [22].

Despite the countless scientific works that accurately describe these processes in living subjects, in the postmortem era, these still remain very limited.

The purpose of this review is to conduct a systematic analysis of the most recent and relevant literature on the potential utility of evaluating the degradation of diverse mRNA types as a new tool for a more exact assessment of the PMI.

## 2. Materials and Methods

### 2.1. Search Strategy

An evidence-based systematic review of the literature has been conducted according to the 2020 PRISMA guidelines, evaluating the main and most recent studies that try to propose a new tool for a more precise postmortem interval estimation based on the degradation times of various RNAs. The search has been performed making use of the electronic databases PubMed and Scopus. The search has then been extended by checking the reference lists of the articles principally eligible for inclusion. A pre-selection of the enrolled articles has been carried out following specific inclusion criteria, such as the full-text availability, the English language, and the year of publication before 2013. Duplicates have been removed. Some articles have been excluded based on titles and abstracts considered not relevant to the review.

The following search queries were used: (i) RNA degradation and PMI estimation; (ii) mRNA degradation and PMI estimation; (iii) mRNA degradation and postmortem interval estimation; (iv) mRNA degradation and time since death and forensic pathology; (v) mRNA degradation and early PMI estimation; (vi) ((mRNA degradation) AND (PMI estimation)) OR (postmortem interval estimation). The last date of the search was 12 June 2024. According to the PRISMA guidelines, the enrolled articles have been separately evaluated by three of the authors.

### 2.2. Data Extraction

A total of 417 articles from the electronic databases PubMed and Scopus were identified. After the removal of duplicates, and of all those articles that did not fit with the aim of the review, 21 articles were enrolled. In particular, the first selection was based on titles and abstracts. Then, all those articles with no full-text availability and not written in English were excluded. Data extraction was performed by two investigators; the data were then verified by three more authors. According to the aim of the paper, a table indicating the extrapolated data of the enrolled articles has been structured. In particular, the authors focused on the type of mRNA, sample, sample storage temperature, PMI time frame analyzed, PMI significance, PMI epicrisis, the use of reference control gene/DNA/RNA, statistical analysis, and estimated error. These data offer an overview of the main heterogeneities related to each study (i.e., studied mRNA, analyzed temperature) and sample characteristics (i.e., human or animal, number of cases, type of tissue). Furthermore, data about the use of the reference control gene/DNA/RNA, statistical analysis, and estimated error have been reported to describe both the robustness or the weak points of the results reported in the papers, also evaluating the risk of bias. The systematic review was not registered in a public registry.

## 3. Results

**Figure 1:**
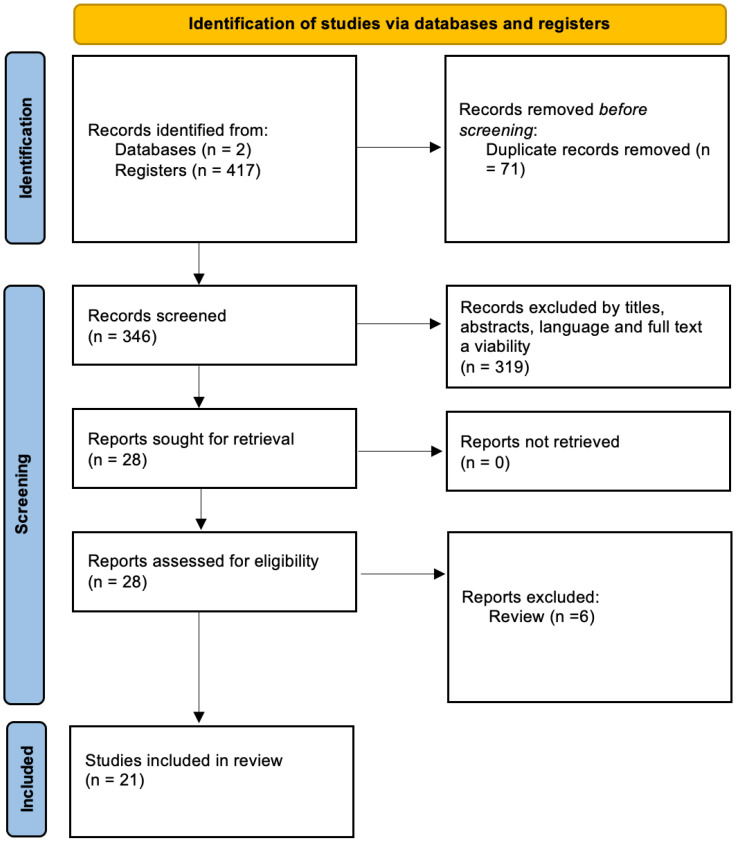
Flowchart of 2020 PRISMA guidelines.

In the literature, some studies trying to identify specific mRNA for more precisely estimating the PMI have been proposed. Figure 1 shows the articles’ selection results. The selection process led to the enrollment of 21 articles, including original articles, short communications, case reports, and case series.

### 3.1. Risk of Bias

This systematic review has some strong points, such as the detailed flowchart describing the study selection process. Despite that, it must be highlighted that this review includes studies that have been published in a time frame of 11 years, as the authors focused on the most recent articles. Furthermore, the obtained data could have an intrinsic risk of bias due to the different analysis tools that have been used by each research group. Because of the heterogeneities related to the type of study (i.e., different mRNAs, analyzed temperature), sample characteristics, and the lack of the estimated error in several studies, the data require further confirmation.

### 3.2. mRNA and PMI Estimation

Deng W. et al. [23] analyzed the degradation pattern of HIF1-α and β-actin mRNAs on a population of 48 mice sacrificed by cervical dislocation after isoflurane anesthesia. The sample was divided into two groups, based on temperature (4 and 37 °C). The analysis of mRNA degradation was conducted on the brain, heart, striated muscle, and liver, sampled at previously established PMIs (0, 1, 2, 6, 12, 24, 36, 48 h). Because of the contradictory results obtained in their previous study, the corresponding DNA of each mRNA was used for the normalization. The obtained results allowed us to appreciate the high stability of both HIF1-α and β-actin mRNAs at 4 °C, not making them useful for PMI estimation within 48 h, while at 37 °C, both mRNAs remained stable during the first two hours (PMI: 2 h), rapidly decreasing from 2 to 24 h in both cerebral and cardiac tissues. Therefore, the authors concluded that the analysis of Hypoxia-inducible factor 1- alfa (HIF1-α) and β-actin mRNA degradation at 37 °C could be a useful tool for estimating the PMI between 2 and 24 h after death.

Sampaio-Silva F. et al. [24] analyzed the degradation pattern of 11 RNAs to develop a mathematical model for estimating the PMI on a mouse model. A population of 15 rats was enrolled, anesthetized, and sacrificed by cervical dislocation: each rat underwent sampling on the heart, femoral quadriceps, and liver at pre-established PMIs (1, 4, 10 h). The samples were stored at a temperature of 21 °C. Among the RNAs, β-actin, glyceraldehyde-3-phosphate dehydrogenase (GAPDH), Hypoxanthine Phosphoribosyltransferase

(HPRT), cytochrome P4502E1 (Cyp2E1), and Peptidylprolyl Isomerase A (Ppia) were selected for their stability, albumin (Alb) and Betaine--Homocysteine S-Methyltransferase (Bhmt) as liver-specific, and myosin light chain kinase (Mylk) and Tropomyosin alpha-1 chain (Tpm1) as femoral quadriceps and cardiac muscle-specific. The 40S ribosomal protein S29 (Rps29) and the 72 kDa subunit of the signal recognition particle (Srp72) genes were chosen as newly studied RNAs. The decay of several femoral quadriceps and liver transcripts positively correlated with the PMI, showing a great variation in the delta-Ct values. Conversely, no statistically significant correlations were obtained between the degradation times studied on the heart and the PMI. The developed mathematical model showed a confidence interval of ±51.4 min.

Young S.T. [25] sacrificed and buried eight pigs, from each of which they randomly took two teeth, establishing a maximum observation time of 140 days after death. The samples were taken at 0 h, 7 d, 14 d, 21 d, 28 d, 42 d, 56 d, 70 d, 84 d, 98 d, 112 d, 126 d, and 140 d. RT-PCR was used to evaluate the degradation times of the mRNA. The RNA extraction and reverse transcription to cDNA were performed; then two sets of porcine-specific RNA primers and probes (PBA71 and PBA301) were created. Two different decay rates of β-actin mRNA were obtained: the former referring to the larger and labile segment (PBA301), the latter to the smaller and more stable non-overlapping segment (PBA71). At the same time, colorimeter assay was used to evaluate alterations in pulp tissue: by combining colorimeter assay and RT-PCR, a new model for calculating the PMI, valid for up to 84 days, was proposed.

Pan H. et al. [26] enrolled a population of 18 rats, randomized into three groups based on temperature (4, 15, 35 °C) to analyze the timing of degradation of five different RNAs: GAPDH and β-actin mRNA, miR-203, 18S rRNA, and 5S rRNA. The analysis was conducted on skin samples taken between 0 and 120 h after death in each group. The results were analyzed comparatively: 5S rRNA and miR-203 proved to be the ones with greater stability, and therefore, are the most suitable as internal controls. β-actin and GAPDH mRNA displayed a good linear relationship at 4 and 15 °C, while at 35 °C, they showed a sigmoid curve. 18S rRNA showed a partial linear relationship with the PMI at both 15 °C and 35 °C. The authors concluded that the rat skin could represent an excellent matrix on which to study RNA degradation, with reference to GAPDH and β-actin mRNA.

In 2014, Lv et al. [27] analyzed the postmortem degradation times of 18S rRNA, U6 small nuclear RNA (snRNA), β-actin 1 and 2, GAPDH1 and GAPDH2 mRNAs, and miR-125b and miR-143 microRNAs, on the spleen of 18 rats. The sample was randomized into two groups: the first group, composed of 12 rats, was further randomized into two subgroups based on the storage temperatures (4 and 25 °C): in the first subgroup, the samples were taken at 0, 1, 3, 6, 12, 24, 36, 48, 72, 96, 120, and 144 h, and in the second, they were taken at 0, 12, 24, 36, 48, 72, 96, 120, 144, 168, 192, 216, 240, 264, 288, and 312 h after death. To validate the model, a third group composed of six rats was included and subsequently randomized into two further subgroups stored at the same temperatures: in the first subgroup, the samples were taken at 0, 5, 55, and 105 h, and in the second, they were taken at 0, 20, 100, 180, and 260 h after death. After validation, mir-125b and mir-143 microRNAs were identified as endogenous control markers, as they were less affected by both the temperature and PMI. The following results have been obtained: at 25 °C, β-actin1 and GAPDH1 showed a slight cubic curve, while β-actin2 and GAPDH2 showed a rapid decrease; thus, β-actin2 and GAPDH2 were considered useful in determining the early PMI. In contrast, at 4 °C, the results showed lower precision. On the other hand, 18S rRNA and U6 snRNA showed an inverse trend; at 25 °C, 18S rRNA showed a parabolic trend, increasing at 4 °C. An opposite trend was recorded for U6 snRNA. 18S rRNA and U6 snRNA were, therefore, considered useful for estimating the “late” PMI.

Sharma et al. [28] proposed a comparative analysis between the apoptosis antagonizing transcription factors (AATF) mRNA and miR-2909, evaluating their postmortem variations in relation to the circadian rhythm. Nine adult balb/c mice were enrolled and maintained at a controlled temperature of 25 °C before being sacrificed. The mice were randomized into three groups, based on the time of death (at 4 am, 8 pm, and 12 pm). To evaluate the stability of AATF mRNA and miR-2909, the carcasses were observed for up to 72 h; samples taken from the kidneys, liver, spleen, pancreas, brain, heart, and lungs were analyzed. The results obtained allowed us to obtain a statistically significant correlation between the variation in AATF mRNA and miR-2909 levels and the circadian rhythm. In particular, AATF mRNA levels remained stable up to 24 h if the mice were sacrificed at 8:00 pm and up to 12 h if sacrificed at 12:00 pm; whereas miR-2909 levels remained stable up to 48 if mice were sacrificed at 8:00 pm and up to 12 h if sacrificed at 12:00 pm.

Ma J. et al. [29] focused on the degradation time of Let-7a, 18S rRNA, miR-125b, miR-9, GAPDH, RPS29, 5S, β-actin, and U6 snRNA, analyzed on a sample of 270 rats killed by cervical dislocation. The study was performed on brain tissue. The enrolled sample was randomized into five groups: the first group (PMI at 0 h) was used as a control, the other four were further divided according to both PMIs (1, 3, 6, 12, 24, 36, 48, 72, 96, 120, and 144 h) and the temperatures at which the sample was stored (4, 15, 25, 35 °C). Another group of 36 rats was used for normalization: the samples were taken at 10, 30, 50, and 100 h and subsequently stored at 10, 20, and 30 °C. The results showed a high tissue specificity and stability of mir-9 and mir-125b, making them useful tools for estimating the PMI up to 144 h. Among other tested markers, β-actin (dCT) highlighted a clear reduction as the postmortem interval increased and, therefore, was used to produce and validate a mathematical model, with a low error percentage, ranging from 30% (30 h at 20 °C) to 43% (10 h at 30 °C). Therefore, brain-specific mir-9 and mir-125b are excellent endogenous controls, as they remain stable up to 144 h after death, at temperatures between 10 and 35 °C.

In 2016, Lu Y.H. et al. [30,31] conducted two studies to establish a mathematical model predictive for PMI estimation. They analyzed the degradation patterns of eight different RNAs (β-actin, GAPDH, RPS29, 18S rRNA, 5S rRNA, U6 snRNA, miRNA-9, and miRNA-125b), using 5S rRNA, miRNA-9, and miRNA-125b for normalization. In both studies, the authors chose the brain as the tissue of interest. The first study (30) was conducted on a mouse model, enrolling a sample of 222 rats subsequently randomized into five groups, respectively, i.e., a control group (PMI 0 h) and four further groups, performing serial brain samples at 1, 2, 4, 6, 8, 10, 12, and 24 h after death. The four groups were stored at different temperatures (5, 15, 25, and 35 °C). The second study (31) was instead conducted on a human model, analyzing the same RNAs on the brain tissue of 12 cadavers, with a known PMI between 4.3 and 22.5 h. In both studies, the results allowed us to highlight statistically significant correlations between both β-actin and GAPDH mRNA and the PMI, especially in the first 24 h; in fact, both mRNAs progressively degrade as the PMI increases. Following validation, the average error rates in rat brain tissue using β-actin and GAPDH were 14.1% and 22.2%, respectively, and 24.6% and 41.0% in human brain tissue. Conversely, miRNA-9 and miRNA-125b showed stability during the first 24 h, proving to be suitable as internal reference markers of both rats and human brain tissue.

Poòr V.S. et al. [32] collected 62 healthy, non-carious premolar and wisdom teeth from human volunteers who underwent orthodontic intervention. The aim was to evaluate the degradation time of both β-actin mRNA and 28S-rRNA. Immediately after extraction, the teeth were placed in plastic bags and kept at a controlled temperature of 22–25 °C. The maximum observation time was 121 days. An initial analysis was conducted by evaluating the RNA integrity number (RIN), obtaining statistically significant values within the first 21 days. RT-PCR analysis was subsequently performed on three fragments of different sizes of the aforementioned RNAs (800, 400, and 200 base pairs); this analysis allowed us to extend the time frame of PMI estimation to an interval between 20 and 42 days after extraction. Therefore, despite using only non-decayed teeth and simultaneously performing RIN and RT-PCR analyses of β-actin mRNA and 28S-rRNA, Poor et al. have proposed a new promising tool for PMI estimation.

In another study by Lv Y. et al. [33], a sample of 216 rats was enrolled to analyze the degradation times of the following RNAs: miR-195, miR-200c, 5S, U6, and RPS29 in lung tissue and miR-1, miR-206, 5S, and RPS29 in skeletal muscle. ACTB and GAPDH have been studied, and they showed statistically significant associations with the PMI; therefore, they have been used as reference genes. Notably, the △Ct values of both ACTB and GAPDH increased faster in the lung than in muscle tissue at the same temperature. The study was conducted by enrolling three different groups: for the first group, the samples were randomized into three sub-groups based on the sample storing temperature (10 ± 1 °C, 20 ± 1 °C, and 30 ± 1 °C). Samples were taken at 0, 1, 3, 6, 12, 24, 36, 48, 72, 96, 120, and 144 h after death. Furthermore, to validate the model, a second group of 15 rats was enrolled and then randomized into 5 sub-groups in relation to the storage temperature (10, 15, 20, 25, 30 °C); the samples were conducted at three different intervals, 10, 60, and 110 h after death. Finally, to evaluate the behavior of these RNAs on a human model, a third group of 12 human cadavers, the time of death being between 7 and 73 h from the first observation, was enrolled: in this sample, the temperature was not controlled, so the corpses remained for variable times at ambient temperature, being subsequently transferred to a cold room or directly subjected to autopsy. Samples were taken from the upper lobes of the left lung and the quadriceps femoris of the right leg. The study demonstrated how a multi-temperature, multi-index mathematical model could increase the accuracy of PMI estimation, resulting in an error rate of 7.4% for rats and 12.5% for humans.

During the following year, Lv Y. et al. [34] performed another study to evaluate the degradation times of some miRNAs, also in humans, analyzing the potential role of mRNAs as reference markers. A total of 13 cadavers with known PMIs varying between 6 and 71 h were selected; samples were taken from the myocardial tissue (apex cordis), from the right lobe of the liver, and from the frontal cortex of the brain. The samples were kept at 4 °C, 15 °C, 25 °C, and 35 °C. miR-1, miR-133a, miR-122, miR-9, and miR-125b were selected as the miRNAs of interest, along with 18S and 5S rRNAs. β-actin, GAPDH, and RPS29 mRNA were used as reference RNA for normalization. miR-133a and miR-1 demonstrated high stability in the muscle myocardium for more than five days at all temperatures. In contrast, miR-122 degrades along the PMI in the liver, especially at high temperatures. They concluded that miR-1 and miR-133a can act as markers or reference genes in the heart because they have high stability, while miR-122 cannot be used as a reference gene in the liver due to its instability and rapid degradation.

Bai X. et al. [35] enrolled a population of 29 rats sacrificed by cervical dislocation to analyze the degradation pattern of Hypoxia associated factor (HAF) mRNA on brain tissue over a period of 48 h after death. Caspase-3 DNA was used to normalize the HAF mRNA degradation. The results obtained made it possible to highlight a progressive increase in HAF mRNA throughout the entire observation period. This increase was greatest in the time interval between 0.5 and 4 h after death. Therefore, this interval was the one in which greater significance was highlighted for the PMI estimation.

Elghamry H.A. et al. [36] analyzed the degradation pattern of GAPDH mRNA on the brain tissue of 78 female rats, randomized into five groups. The first group was used as a control: six rats were included, and the samples were taken at time 0 (PMI 0 h). The other four groups consisted of 18 rats each, stored at 30 °C in the open air, buried in sand at 30 °C, submerged under water at 30 °C, and stored in a refrigerator at a temperature of 6 °C, respectively. For each group, three brain tissue samples were taken at 24 h, 48 h, and 96 h after death. β-actin mRNA was used as a reference gene for normalization. The analysis of the results made it possible to highlight the highest degradation rate after 96 h in the first group, where the population remained in ambient air at 30 °C. Conversely, the lowest degradation rate was observed in group four, where rats were stored at 6 °C. The authors concluded that GAPDH mRNA on brain tissue can be used to estimate the PMI and that its degradation is influenced by temperature and is more rapid at a higher temperature.

Ali M.M. et al. [37] analyzed the degradation times of the late cornified envelope 1C (LCE1C) mRNA on 12 skin tissue fragments collected from six living humans during surgery. The 12 skin fragments were divided into two groups depending on the two different temperatures at which they were stored, 24/25 °C and 40 °C, respectively. Subsequently, six different samples were taken at 0 h, 1 d, 2 d, 3 d, 4 d, and 5 d. The LCE1C mRNA values were quantified by RT-PCR, using GAPDH mRNA as an endogenous reference housekeeping gene. The study allowed us to document a progressive, time-related reduction in LCE1C mRNA, considered statistically significant up to 5 days after death. No differences in the LCE1C mRNA degradation times were observed between the two groups; therefore, the temperature was not considered a relevant factor.

Fais et al. [38] conducted a study on 10 cadavers and used the gingival tissue, taken during autopsy from maxillary gingiva, at a known PMI. The sample was randomized into three different groups based on the PMI: a short PMI (SPMI) from 1 to 3 days, a medium PMI (MPMI) at 4–5 days, and a long PMI (LPMI) at 8–9 days. The aim was to evaluate the degradation times of HIF-1α protein and its mRNA at different PMIs. Both immunohistochemical analysis and RT-PCR analysis were performed simultaneously. A progressive increase in the levels of both HIF-1α protein and its mRNA was observed at the SPMI and MPMI, being greater at the SPMI, while at the LPMI, neither positive stained areas involving immunohistochemistry nor HIF-1a mRNA expression were detected. Therefore, statistically significant results, obtained by integrating the PMI-related variation values of HIF-1α protein and its mRNA, were obtained for the SPMI and MPMI but not for the LPMI.

Tao L. et al. [39] conducted a pilot study with the aim of identifying a reliable mRNA for an early PMI estimation; the study was conducted on the cardiac tissue of 91 rats, stored at three different controlled temperatures, 10, 25, and 35 °C, respectively. Each sampling was performed at 0, 1, 3, 6, 12, 24, and 36 h after death. To verify their new mathematical model, another 27 rats were sacrificed and stored at 25 °C, 30 °C, and 35 °C: the estimated error rate was less than 15%. Of the 217 markers studied, cell division cycle 25 homolog B mRNA (Cdc25b) has been found to be the most sensitive for ePMI estimation. The quantitative Cdc25b mRNA temporal variation was decreased at 25 °C from 0 to 6 h and increased from 6 to 18 h, whereas at 35 °C it was increased from 0 to 18 h. Rpl27 mRNA was used for normalization.

Alshehhi S. et al. [40] comparatively analyzed the degradation times of five different mRNAs and miRNAs for an overall period of one year, evaluating their variations at defined intervals (0, 7, 14, 28, 90, 180, 270, and 360 days). The analysis was performed on two different matrices, represented by the saliva and semen of 19 voluntary living donors. After collection, the sample was stored at room temperature. The RNAs studied were Statherin (STATH) mRNA and miR205 on saliva and PRM1 mRNA and PRM2 mRNA, miR891a, and miR10b on semen. Actin beta (ACTB),18S, and U6 were used as reference genes for normalization. The results obtained showed the high stability of miR891a and miR205 up to 360 days, whereas miR10b remained stable up to 14 days, STATH mRNA up to 28 days, and protamine 1 (PRM1) and protamine 2 (PRM2) mRNA up to 90 days. The authors concluded that miRNA markers have greater stability, probably due to their small size, while Cq values for mRNAs tend to increase over time, indicating their degradation due to their greater instability.

Peng D. et al. [41] conducted an analysis on a sample of 87 mice, observing the degradation time of five different mRNAs in a range between 0 and 48 h after death, performed on cardiac and brain tissue. Hypoxia-inducible factor 2 alpha–short fragment (HIF2a-S), hypoxia-inducible factor 2 alpha–long fragment (HIF2a-L), HAF, apoptosis-inducing factor (AIF), and factor inhibiting HIF (FIH) mRNAs were studied. Caspase-3 DNA and 18S were used for normalization. mRNA and DNA were extracted simultaneously within the same tube. The following results were obtained: in cardiac tissue, HIF2a-S, HIF2a-L, AIF, and FIH showed a statistically significant correlation with the PMI within 48 h, after normalization with both Caspase-3 DNA and 18S. No correlation was found for HAF in the heart tissue. In the brain tissue, significant correlations were found for HIF2a-S, HIF2a-L, and HAF when normalized with 18S and for HIF2a-S, HIF2a-L, HAF, FIH, and AIF when normalized with Caspase-3. HIF2a-S showed a greater statistical significance than HIF2a-L, highlighting that shorter mRNA fragments are more suitable than longer ones. In heart tissue, HIF2a-S, HIF2a-L, AIF, and FIH decrease within 48 h after death, whereas, in brain tissue, HIF2a-S, HIF2a-L, AIF, and FIH decrease, while HAF increases. It has also been demonstrated that DNA and mRNA can be successfully coextracted in one tube.

Wang Y. et al. [42] conducted a study on three domestic pigs, which after being sacrificed, were immediately placed in a mountain meadow. The observation period had a total duration of 240 h. The samples, carried out on skeletal muscle, were performed every two hours for up to 10 h and every 24 h starting from the following day. During the observation period, the temperature and humidity were measured every hour and varied from a minimum of 16.08 to a maximum of 26.25 °C. Three different mRNAs were examined: Peptidylprolyl isomerase A (Ppia), glyceraldehyde-3-phosphate dehydrogenase (GADPH), and β-actin. The expression levels of both PPIA and GAPDH genes increased in the first 10 h and then gradually decreased from 10 to 240 h. On the contrary, β-actin gradually decreased from the first observation to 240 h. The analysis performed allowed us to identify a statistically significant association between the variation in the expression of these three genes within the first ten hours and the PMI, while their reliability progressively reduced from 10 to 240 h.

Wang Y. et al. [43] proposed another study for the evaluation of the degradation times of some mRNAs in rat carcasses kept in water. The analysis was conducted on a sample of 45 rats, killed by cervical dislocation, and placed in a river inside plastic net bags, for 18 days. During the entire observation period, environmental temperature values were monitored (mean air temperature 22.8 °C; lowest 18.8 °C, highest 25.9 °C), including the water temperature (mean water temperature 20 °C; lowest 19.5 °C, highest 20.2 °C). The samplings were performed on the rat brain tissue, at regular intervals: 0 (0 h), day 1 (24 h), day 2 (48 h), day 3 (72 h), day 4 (96 h), day 5 (120 h), day 6 (144 h), day 7 (168 h), day 8 (192 h), day 9 (216 h), day 10 (240 h), day 12 (288 h), day 14 (336 h), day 16 (384 h), and day 18 (432 h). The analysis was conducted by analyzing the degradation pattern of β-actin, GADPH, and 18S mRNAs, while 5S was used as a reference gene. The studied mRNAs presented different degradation patterns: β-actin was upregulated from 0 to 6 days, proving to be the most promising of all for estimating the PMI; GADPH increased on the first day, was then downregulated on the second day, and increased again from 3 to 6 days; finally, 18S decreased during the first day, gradually increased from the second to the fifth day, and decreased again on day 6. Therefore, the authors concluded that the analysis of the brain tissue of all three enrolled mRNAs, when normalized with 5S, proved to be a promising tool for PMI estimation for up to 6 days in carcasses kept in an aqueous environment.

Guardado-Estrada M. et al. [44] conducted a study on miRNA expression variation in rat skeletal muscle within 24 h of death (early PMI). Nine rats were enrolled, kept at a temperature of 22 °C, and subsequently randomized into two groups based on the PMI: four constituted the control group (0 h PMI) and five the other group (24 h PMI). A total of 1.218 rat miRNAs were analyzed using the Affymetrix GeneChip miRNA 4.0 Array probes of mature rat miRNAs. A total of 156 miRNAs underwent changes within 24 h, resulting in 84 downregulated and 72 upregulated. Among these, miR-139-5p was found to be the most significantly downregulated, while rno-miR-92b-5p was the most significantly upregulated. In the study, interactions between miRNAs and mRNAs were also observed: interactions with 130 different mRNAs were found for rno-miR-125b-5p/rno-miR-351-5p, while 120 were found for rno-miR-138-5p. The gene expression of transforming growth factor beta receptor 2 (TGFBR2), Sirtuin 1 (SIRT1), and Bcl-2-modifying factor (BMF) mRNAs, which are, respectively, the targets of rno-miR-291a-3p, rno-miR-125b-5p/rno-miR-351-5p, and rno-miR-138-5p, were subsequently analyzed with qRT-PCR. These mRNAs were found to take part in postmortem autophagy, cell cycle regulation, and the low oxygen response. SIRT1 mRNA was found downregulated at 24 h, and TGFBR2 and BMF mRNAs were upregulated. Statistically significant associations between the mRNA degradation time and the PMI were found for SIRT1 and TGFBR2 but not for BMF.

The main results of the enrolled articles are reported in Table 1, Table 2 and Table 3. The tables were structured based on the assessed time frame.

## 4. Discussion

Trying to estimate the PMI as precisely as possible is one of the most important challenges that forensic pathologists have to daily deal with in carrying out their activities. Therefore, in recent decades, research has heavily focused on molecular diagnostics [44]. Among the investigations under development, one of the most promising is represented by the evaluation of the degradation times of RNA molecules, and, among these, mRNAs have been one of the most studied, especially in the estimation of the early PMI [30,37,39,45,46,47].

### 4.1. Data Normalization and Mathematical Models

For a correct analysis of RNA molecule degradation times, it is essential to use methods that can quantitatively analyze variations within defined time frames [48]. Quantitative real-time polymerase chain reaction (qRT-PCR) is currently considered the method of choice [48]. However, it is known that tissue sampling can cause alterations in the real concentrations of RNAs, leading to changes in gene expression. The inevitable consequence would be incorrect data interpretation. For these reasons, proper data standardization is deemed critical [49]. For this reason, if research evaluating RNA degradation is conducted, it is essential to use reference genes for normalization [50]. Reference genes are internal reaction controls with sequences that differ from the target. To be considered a reliable reference, a gene must meet several strict parameters [50]. Among these parameters, the most crucial is considered to be its expression level, which has to remain unchanged under experimental conditions [51].

GAPDH, β-actin, RPS-29, and IL-1β are nowadays considered effective endogenous reference genes, being among the most used in qRT-PCR data analysis in general molecular biology experiments [52]. Despite that, these markers tend to degrade over time, especially under risky environmental circumstances, such as high temperatures, losing their reliability as PMI indicators. Therefore, significant limitations still affect these methods, primarily due to the difficulty in standardizing quantitative analyses, not always managing to make them sufficiently precise and reproducible [53]. To further reduce the error percentage linked to incorrect normalization, the geNorm and Normfinder algorithms have defined a group of reference genes, specific for each tissue and to be used simultaneously to further increase the accuracy of the investigations [52].

Another fundamental aspect of limiting errors attributable to incorrect data understanding and interpretation is represented by the use of mathematical models [54]. It is now known that building mathematical models simplifies the identification of confounding factors that occur during the analysis of complex data, which would inevitably influence the research results (54). Therefore, building a mathematical model would enable us to achieve both precise predictions and the correct quantification of relationships between several variables, refining data interpretation of large datasets and reducing the error rate [55].

Lv Y. et al. [33,34] underlined how the use of mathematical models is of fundamental importance in reducing the error rate and making the results more reliable. Thanks to the use of their mathematical model, they were able to reduce the error rate to 7.4% for rats and 12.5% for humans in the first study [33] and to 2.89% in rats and 5.06% in humans in the second one [34], demonstrating how a multi-temperature, multi-index mathematical model could increase the accuracy of PMI estimation.

### 4.2. Main Evidence on mRNAs in PMI Estimation

It has been said that most of the studies that have evaluated the use of mRNA molecules to achieve a more precise estimate of the PMI have been conducted over narrow time intervals, often not reaching observation times longer than one week [23,24,26,28,29,30,31,33,34,35,36,37,38,39,41,42,43,44]. Among the various mRNA types, β-actin and GAPDH have undoubtedly been the most studied [23,24,25,26,27,29,30,31,32,33,34,36,42,43]. In most cases, the temporal variation in the concentration of the two molecules was analyzed simultaneously [24,26,27,29,30,31,34,42,43], while the cases in which exclusively β-actin [23,25,32] or GAPDH [33,36] were analyzed were rarer. Among the other mRNA molecules analyzed, HIF1α mRNA [23,38] and HAF mRNA [35,41] were of particular interest.

It is equally important to underline that, in numerous studies, different types of molecules have been enrolled for normalization [23,24,26,27,29,30,31,33,34,35,36,37,38,39,40,41,43,44]. Among these, the first category is represented by microRNAs [27,29,30,31]: microRNAs are molecules that have high and stable expression levels, thus being particularly useful for this purpose [56]. DNA [23,35,41] and ribosomal RNA [27,30,33,40,41,43] molecules have also been used. It is interesting to underline that, in some studies, β-actin and GAPDH were also used for normalization [36,37,38,44].

β-actin mRNA encodes the β-actin protein, which is an extremely important component of the eukaryotic cytoskeleton, essential for structural support, cell motility, division, and intracellular transport [57]. It is known that its gene, ACTB, is highly conserved and ubiquitously expressed across various cell types, making β-actin mRNA a common housekeeping gene in gene expression studies [57]. This consistent expression allows it to serve as a reliable reference for normalizing data in techniques like qPCR and Northern blotting [58]. In research, β-actin mRNA helps ensure accurate and reliable gene expression measurements by normalizing target mRNA levels and correcting for sample quantity and quality variations [58]. However, it is crucial to verify its stable expression under experimental conditions, and this is why multiple housekeeping genes, such as GAPDH and 18S or 5S rRN, are often used for an accurate normalization [26,27,33,37].

GAPDH mRNA encodes glyceraldehyde-3-phosphate dehydrogenase, a pivotal enzyme in glycolysis, and it is frequently used as a reference gene for normalizing data across diverse conditions, typically expressed at relatively constant levels across different cell types and conditions [59]. Despite that, especially in forensic settings, several factors capable of influencing its degradation have been described, especially the environmental temperature [23,24,36,59]. Temperature is known to influence RNA degradation patterns, generally increasing it at higher temperatures and reducing it at lower ones. This is due to the temperature sensitivity of the enzymes that deal with their degradation (RNases) [60]. Therefore, in research settings, controlling temperature is essential for trying to standardize the mRNA degradation pattern and create a shareable model [60]. Deng W. et al. [23] analyzed the degradation pattern of HIF1-α and β-actin mRNAs at 4 and 37 °C over a period of 48 h, detecting statistical significance only at 37 °C. Similar results were obtained by Lv Y. et al. [27] for β-actin mRNA and GAPDH analyzed at a temperature of 25 °C, while at 4 °C, no elements of statistical significance were found. Furthermore, a lower error rate was detected at 25 °C (<10%), compared to 4 °C. Anyway, in both cases, at low temperatures, there was a slowdown in the degradation times of the mRNAs of interest. Therefore, the authors concluded that these molecules have greater reliability in PMI estimates at higher temperatures. Likewise, Ma J. et al. [29] investigated the degradation times of β-actin and GAPDH mRNA at 20 and 30 °C, finding greater stability at the lower temperature; considering the rapid degradation of β-actin, it was used to produce and validate their mathematical model. Considering the degradation times of β-actin and GAPDH mRNA, most authors agree in considering them useful for estimating the PMI, especially within the first seven days after death and within the first 48 h, if kept at temperatures between 20 and 35 °C [23,24,26,27,29,30,31,33,34,36,42,43]. Studies conducted on other RNAs, such as HIF-1α mRNA, have also shown agreement on the use of mRNAs within more limited time periods, within 7 days [23,38].

Despite what has just been considered, Young S.T. et al. [25] and Poor V.S. et al. [32] proposed the use of β-actin mRNA for much longer time intervals, up to 84 and 42 days, respectively, after death. Both studies were conducted on the dental pulp of pigs, in the first case, and on human volunteers who underwent orthodontic intervention, in the second case. Young S.T. et al. [25] analyzed the timing of the degradation of two β-actin fragments, PBA301, which is the larger and labile segment, and PBA71, which is the smaller and more stable non-overlapping segment. The smaller fragment proved to be more stable and, therefore, more reliable in the case of longer time intervals. Poor V.S. et al. [32] instead proposed the evaluation of β-actin mRNA degradation using two different methods: the RNA Integrity Number and qRT-PCR. RIN is a numerical assessment used to evaluate the integrity and quality of RNA samples, ranging from 1 to 10, with higher numbers indicating better RNA integrity and quality [61]. Conversely, qRT-PCR quantifies the expression levels of specific mRNA targets [48]. The evaluation of the RIN allowed us to obtain statistically significant values within the first 21 days, while the qRT-PCR analysis, subsequently performed on three fragments of different sizes (800, 400, and 200 base pairs), allowed us to extend the time frame of PMI estimation to an interval between 20 and 42 days after extraction. Therefore, the simultaneous use of different methods can increase the accuracy of the analysis. Alshehhi S. et al. [40] also proposed the use of mRNA molecules for the evaluation of intermediate–long PMIs. The analysis of STATH mRNA on saliva and PRM1 mRNA and PRM2 mRNA on sperm proved the usefulness of STATH mRNA for up to 28 days and PRM1 and PRM2 mRNA for up to 90 days.

Therefore, the same type of RNA (mRNA) or even the same molecule, such as β-actin and GAPDH mRNA, are capable of behaving differently, making them potentially useful in very different time intervals, ranging from a few hours to several weeks or months [23,32,40]. In fact, the degradation time of the same type of mRNA, in addition to being influenced by external factors such as environmental temperature, is inevitably influenced by other factors, such as the specific type of tissue in which it is observed [18].

Currently, the most used matrices include the brain [28,29,30,31,34,35,36,41,43], heart [24,28,34,39,41], and skeletal muscle [24,33,34,42]. Other tissues are instead represented by skin [26,37], dental pulp [25,32], spleen, lungs, and gingival tissue [24,27,28,33,34,40]. One of the reasons why the brain and heart are among the preferred organs for this type of research is that they are more protected and, therefore, less exposed to the external environment [62]. In fact, both the skull and the brain, as well as the rib cage and the pericardial sac for the heart, allow them to be less subject to external factors, such as the environmental temperature, reducing some of the biases previously considered [44]. Similar considerations are valid for dental pulp [25,32]. Another reason why the brain, heart, and dental pulp are preferred to other internal organs is that they contain a smaller quantity of ribonuclease, and being more protected, they are also less exposed to the action of pathogens (e.g., bacteria), which contain exogenous ribonucleases [52].

Despite these aspects, studies conducted on skin samples have also allowed the finding of statistical significance between the degradation times of specific mRNAs and PMI [26,37]. Ali et al. [37] proposed the analysis of a new mRNA, LCE1C mRNA. They observed not only its progressive degradation as the PMI increases but also an absence of significant modifications in its temperature-related degradation times (24/25 and 40 °C).

### 4.3. Limitations

Ultimately, despite the data available, there are still numerous limitations that make these studies inaccurate and difficult to reproduce: (i) most of the articles present in the literature evaluate the degradation times of a limited number of mRNAs, often within narrow time frames; (ii) much of the research has been conducted on animal models, while there are still too few studies that have managed to accurately reproduce these results on human models; (iii) many of the experiments conducted exclusively provide data on the linear correlation between RNA degradation and an increase in the PMI but do not provide any data on the percentage of error between the two parameters; (iv) only an extremely limited number of studies make use of mathematical models, which are fundamental to further reduce the error rate.

## 5. Conclusions

In recent years, there has been a notable increase in forensic research aimed at identifying new tools to achieve a more precise estimate of the postmortem interval, which is of pivotal importance in the forensic pathologist’s field. In this context, particular attention has been paid to the degradation times of mRNA. Although nowadays the most promising results seem to be those obtained using multi-parametric models, the lack of standardized and globally accepted procedures makes these analyses unreliable.

Furthermore, the degradation times of a specific mRNA are influenced by several factors, such as the environmental characteristics where the sample is stored, the intrinsic tissue characteristics, and the quantity of ribonuclease contained in the examined matrix. Currently, the heart, brain, and striated skeletal muscle appear to be the most suitable for carrying out these analyses. Given these limitations, to obtain more accurate results, mRNA degradation on different matrices and at different temperatures should be evaluated simultaneously, always using reference genes as internal controls and building mathematical models to minimize the error rate. In any case, considering both the still small number of articles published in the relevant literature and the first promising results obtained, it is possible to assert that the evaluation of the mRNA degradation time could represent a promising tool for reaching more precise PMI estimation in the near future.

## Figures and Tables

**Table 1:** Summary of the main data resulting from the articles analyzing mRNA within a time frame of 48 h.

Authors	Year	mRNA	Sample	Tissue	Sample Number	Temperature	PMI–Time Frame Assessed	PMI Significance	PMI Epicrisis	Reference Control Genes/RNA/DNA–No	Statistical Analysis	Estimated Error
Deng W. [23]	2013	β-actin and HIF1α mRNA	Mice	Brain, Heart, liver, skeletal muscle	48	4, 37 °C	0, 1, 2, 6, 12, 24, 36, 48 h	Between 2 and 24 h at 37 °C on heart and brain	Stable up to 48 h at 4 °C; stable during the first 2 h, then decreased at 37 °C	Correspondent DNA	ΔCt method	N.U. *
Sampaio-Silva F. [24]	2013	β-actin, GAPDH, Hprt, Cyp2E1, Ppia, Alb, Bhmt, Mylk, Tpm1, Rps29, Srp72 mRNA	Balb/c mice	Heart, femoral quadriceps, liver	15	21 °C	1, 4, 10 h	Up to 11 h	Medium PMI 1.90 ± 0.01 4.10 ± 0.87 9.80 ± 1.87	Rps29	Linear regression analysis; Pearson correlation	Mean error rate ± 51.4 min
				Skin, heart, spleen, femoral quadriceps, liver, pancreas, stomach, lungs	5		4, 20 h					
Lu Y.H. [30]	2016	β-actin, GAPDH and RPS29 mRNA, 5S and 18S rRNA, U6 snRNA	Rat	Brain	222	5, 15, 25, 35 °C	0, 1, 2, 4, 6, 8, 10, 12, 24 h	β-actin and GAPDH up to 24 h	β-actin and GAPDH degraded as PMI increases	5S rRNA, miR-9, miR-125b	Regression analysis by SPSS software v.19	Average error rate: 14.1% (β-actin) and 22.2% (GAPDH)
		miR-9						miRNA-9 and miRNA-125b remain stable for up to 24	Stable up to 24 h			
		miR-125b										
Lu Y.H. [31]	2016	β-actin, GAPDH and RPS29 mRNA, 5S and 18S rRNA, U6 snRNA, miR-9, miR-125b	Dead human	Brain	12	N.R. *	From 4, 3 to 22, 5 h	β-actin and GAPDH up to 24 h	β-actin and GAPDH degraded as PMI increases	5S rRNA, miR-9, miR-125b	Quadratic regression (SPSS software v.19)	Error rate: 24.6% (β-actin) and 41.0% (GAPDH)
		miR-9						miRNA-9, miRNA-125b and 5S rRNA were suitable as internal reference markers	Stable up to 24 h			
		miR-125b										
Bai X. [35]	2017	HAF mRNA	Rat	Brain	29	N.R. *	from 0.5 to 48 h	from 0.5 to 4 h, accuracy progressively decreases up to 48 h	Increase up to 48 h	Caspase-3 DNA	Linear statistical model	N.R. *
Tao L. [39]	2018	Cdc25b mRNA	Rats	Heart	91	10, 25, 35 °C	0, 1, 3, 6, 12, 24, 36 h	ePMI (0–24 h)	At 25 °C, decreased from 0 to 6 h; increased from 6 to 18 h	Rpl27 mRNA	ΔCt method, Cubic regression	1.23 + 2.51 (25 ° C), 7.12 + 7.62 (30 ° C) and 5.31 + 3.72 (35 °C)
					27	25, 30, 35 °C	6, 12, 18 h		At 35 °C, increased from 0 to 18 h; decreased from 18 to 24 h			
Peng D. [41]	2020	HIF2a-S, HIF2a-L, HAF, AIF, FIH mRNAs	Mice	Brain	87	37 °C	0–48 h	0–48 h	HIF2a-S, HIF2a-L, AIF, and FIH decrease after death; HAF increase after death	Caspase-3 DNA	dCq method	N.R. *
		HIF2a-S, HIF2a-L, HAF mRNAs								18S		
		HIF2a-S, HIF2a-L, AIF, FIH mRNAs		Heart					HIF2a-S, HIF2a-L, AIF, and FIH decrease after death	Caspase-3 DNA, 18S		

* N.R.: Not Reported * N.U.: Not Used.

**Table 2:** Summary of the main data resulting from the articles analyzing mRNA within a time frame of 1 week.

Authors	Year	mRNA	Sample	Tissue	Sample Number	Temperature	PMI–Time Frame Assessed	PMI Significance	PMI Epicrisis	Reference Control Genes/RNA/DNA—No	Statistical Analysis	Estimated Error
Pan H. [26]	2014	β-actin and GAPDH mRNA, 18S, 5S rRNA	Rat	Skin	18	4, 15, 35 °C	From 0 to 120 h	N.R. *	β-actin and GAPDH decrease as PMI increases	β-actin and GAPDH	Regression analysis (GraphPad v.5.0)	N.R. *
		miR-203							Remain stable up to 120 h			
Sharma et al. [28]	2015	miR-2909	Mice	Heart, lungs, brain, spleen, liver, pancreas, and kidneys	9	25 °C	12 h, 24 h, 36 h, 48 h, 72 h	Up to 48 h	stable up to 48 if sacrificed at 8:00 pm; stable up to 12 h if sacrificed at 12:00 pm.	N.U. *	SPSS window v.19 and ANOVA	N.R. *
		AATF mRNA						Up to 24 h	stable up to 24 h if sacrificed at 8:00 pm; stable up to 12 h if sacrificed at 12:00 pm.			
Ma J. [29]	2015	β-actin, GAPDH and RPS29 mRNA, 5S and 18S rRNA, U6 snRNA, miR-9, miR-125b and Let-7a	Rats (Sprague- Dawley)	Brain	270	4, 15, 25, 35 °C	1, 3, 6, 12, 24, 36, 48, 72, 96, 120, and 144 h	N.R. *	(miR-9, miR-125b) up to 144 days	miR-9, miR, 125b	Bivariate cubic curve, quadratic regression	Mean error rate 4.8%. Ranging from 30% (30 h at 20 °C) to 43% (10 h at 30 °C)
					36	10, 20, 30 °C	10, 30, 50, 100 h					
Lv Y. [33]	2016	miR-195, miR-200c, 5S, U6 and RPS29 (lung); miR-1, miR-206, 5S and RPS29 (skeletal muscle)	Rats	Lungs and skeletal muscle	216	10, 20, 30 °C	0, 1, 3, 6, 12, 24, 36, 48, 72, 96, 120, 144 h	Up to 144 h	Stable up to 144 h, then decreased	b-actin and GAPDH, 18S rRNA, RPS29 mRNA	Linear, quadratic, and cubic regression, △Ct method	7.4%
					15	10, 15, 20, 25, 30 °C	10, 60, 110 h					
			Dead human		12	Ambient temperature of the crime scene before being transferred to the freezer or autopsy.	Variable from 7 to 73 h					12.5%
Lv Y. [34]	2017	18S and 5S rRNA, miR-1, miR-133a, miR-122, miR-9, miR-125b	Dead human; rats Sprague-Dawley	Human heart (Apex Cordis), liver (right lobe), brain (frontal cortex)	13 dead humans (7 males, 6 females); 36 rats	4 °C, 15 °C, 25 °C, 35 °C	From 6–71 h up to 5 days	Up to 71 h	Stable up to 5 days	β-actin, GAPDH, RPS29 mRNA	Linear, quadratic, and cubic regression (R software v. 3.6.0), ΔCt method.	Mean estimated error: 5.06 (human); 2.89 (rat)
Elghamry H.A. [36]	2017	GAPDH mRNA	Rat	Brain	78	6, 30 °C	24, 48, 96 h	Up to 96 h	GADPH mRNA decreases as PMI increases; the decrease is greater at higher temperatures, smaller at lower temperatures.	β-actin mRNA	Kruskal–Wallis and Mann–Whitney tests (SPSS v. 22)	N.R. *
Ali M.M. [37]	2017	LCE1C mRNA	Living Human	Skin	12	24/25 °C and 40 °C	0 h, 1, 2, 3, 4, 5 days	Up to 5 days	Progressive degradation as the PMI increases	GAPDH	Linear regression (SPSS v. 21)	N.R. *

* N.R.: Not Reported * N.U.: Not Used.

**Table 3:** Summary of the main data resulting from the articles analyzing mRNA over a period greater than one week.

Authors	Year	mRNA	Sample	Tissue	Sample Number	Temperature	PMI–Time Frame Assessed	PMI Significance	PMI Epicrisis	Reference Control Genes/RNA/DNA–No	Statistical Analysis	Estimated Error
Young S.T. [25]	2013	β-actin mRNA	Pig (8)	Dental pulp	40	Below ground’s surface	0 h, 7 d, 14 d, 21 d, 28 d, 42 d, 56 d, 70 d, 84 d, 98 d, 112 d, 126 d, 140 d	Up to 84 days	N.R. *	N.U. *	ΔCt method	N.R. *
Lv. Y. [27]	2014	β-actin, GAPDH1 and GAPDH2 mRNA, ACTB1 and ACTB2, miR-125b, miR-143, 18S rRNA, U6 snRNA	Rats (18)	Spleen	6	4, 25 °C	0, 1, 3, 6, 12, 24, 36, 48, 72, 96, 120, and 144 h	At 25 °C, miR-125b, miR-143, and U6 up to 36 h; miR-125b, miR-143, and ACTB1 up to 144 h. At 4 °C, miR-125b, GAPDH1, and miR-143 up to 144 h, while miR-125b, miR-143, and ACTB1 up to 312 h	β-actin1 and GAPDH1 slightly decrease, whereas β-actin2 and GAPDH2 depleted rapidly. 18S rRNA has a parabolic-like trend at 25 °C, exponential at 4 °C; Mir-125b Ct values fluctuated slightly within 36 h and then increased slowly at 25 °C; the same trends have been observed within 144 h at 4 °C; U6 snRNA exponentially increased at 25 °C, parabolic-like decline in at 4 °C.	Geometric mean of miR-125b and miR-143 for GAPDH1, GAPDH2, ACTB1, ACTB2, U6, and 18S rRNA; GAPDH1 for GAPDH2; ACTB1 for ACTB2	Linear, quadratic, cubic, reciprocal, exponential, and logarithmic regression (GraphPad v5.0)	<10% at 25 °C and <20% at 4 °C
					6		0, 12, 24, 36, 48, 72, 96, 120, 144, 168, 192, 216, 240, 264, 288, and 312 h					
					3		0, 5, 55 and 105 h					
					3		0, 20, 100, 180, and 260 h					
Poòr V.S. [32]	2016	β-actin mRNA, 28S rRNA	Living human	Dental pulp	62	22–25 °C	0–121 days	0–21 days	* If RNA integrity number (RIN)	N.U. *	Univariate linear regression	N.R. *
								21–42 days	* If RT-PCR			
Fais P. [38]	2018	HIF-1α mRNA	Dead human	Gingival tissues	10	N.R. *	1–3 days; 4–5 days; 8–9 days	1–5 days	1.8-fold upregulation up to 3 days; 3.7-fold upregulation from 4 to 5 days. No HIF-1a mRNA expression detected from day 6.	GAPDH; Hs99999905_m1	GraphPad Prism 5.0 software	N.R. *
Alshehhi S. [40]	2019	STATH mRNA	Living human	Fresh saliva	19	Room temperature	0, 7, 14, 28, 90, 180, 270, 360 days	Stable up to 28 days	Stable up to 28 days, then decrease.	ACTB,18S, U6	Relative expression ratio	N.R. *
		miR205						N.R. *	Stable up to 360 days			
		miR10b		Freshly ejaculated semen				Up to 14 days	Degrade slightly up to 14 days			
		miR891a						N.R. *	Remain stable for up to 360 days			
		PRM1 and PRM2 mRNA						Stable up to 90 days	PMR1 remains stable for up to 14 days, then rapidly decreases; PMR2 progressively decreases during the first 90 days but remains detectable for the entire observation period.			
Wang Y. [42]	2021	β-actin mRNA, GAPDH mRNA, Ppia	Domestic Pigs	Muscle	3	From 16.08 to 26.25 °C	0, 2, 4, 6, 8, 10, 24, 48, 72, 96, 120, 144, 168, 192, 216, 240 h	0–240 h	Linear upward trend within 10 h after death, followed by a gradual downward trend from 10 to 240 h.	N.U. *	ΔCt method, linear regression analysis	N.R. *
Wang Y. [43]	2022	β-actin mRNA	Rats	Brain	45	Mean air temperature 22.8 °C (lowest 18.8 °C/highest 25.9 °C); mean water temperature 20 °C (lowest 19.5 °C/highest 20.2 °C);	0 (0 h), day 1 (24 h), day 2 (48 h), day 3 (72 h), day 4 (96 h), day 5 (120 h), day 6 (144 h), day 7 (168 h), day 8 (192 h), day 9 (216 h), day 10 (240 h), day 12 (288 h), day 14 (336 h), day 16 (384 h), and day 18 (432 h)	Up to 6 days	Upregulated from 0 to 6 days.	5S	Polynomial regression analysis	N.R. *
		GAPDH mRNA							Increase the first day, downregulated on the second day, increase from 3 to 6 days			
		18S							Decreased slightly on day 1, gradually increased from days 2 to 5, and decreased again on day 6			

* N.R.: Not Reported * N.U.: Not Used.

## Data Availability

No new data were created or analyzed in this study.
